# The Computer Will See You Now: Overcoming Barriers to Adoption of Computer-Assisted History Taking (CAHT) in Primary Care

**DOI:** 10.2196/19306

**Published:** 2021-02-24

**Authors:** Pier Spinazze, Jiska Aardoom, Niels Chavannes, Marise Kasteleyn

**Affiliations:** 1 Global Digital Health Unit Department of Primary Care and Public Health, School of Public Health Imperial College London London United Kingdom; 2 Department of Public Health and Primary Care Leiden University Medical Center Leiden Netherlands

**Keywords:** computer-assisted history taking, history taking, clinical consultation, digital health, electronic health record, patient-provided health information

## Abstract

Patient health information is increasingly collected through multiple modalities, including electronic health records, wearables, and connected devices. Computer-assisted history taking could provide an additional channel to collect highly relevant, comprehensive, and accurate patient information while reducing the burden on clinicians and face-to-face consultation time. Considering restrictions to consultation time and the associated negative health outcomes, patient-provided health data outside of consultation can prove invaluable in health care delivery. Over the years, research has highlighted the numerous benefits of computer-assisted history taking; however, the limitations have proved an obstacle to adoption. In this viewpoint, we review these limitations under 4 main categories (accessibility, affordability, accuracy, and acceptability) and discuss how advances in technology, computing power, and ubiquity of personal devices offer solutions to overcoming these.

## Introduction

### Background

Computer-assisted history taking (CAHT) was first explored in medicine in the 1960s as a tool to aid clinicians in gathering data from patients in order to support diagnostic and treatment decisions. Over the years, research has highlighted the numerous benefits of CAHT; however, the limitations have proved an obstacle to adoption. We explore how advances and innovation in digital technology today may offer new solutions to overcoming these barriers.

### The Importance of History Taking in Clinical Care

Collecting an appropriate and comprehensive medical history from patients is a fundamental process in clinical medicine. Research has shown that history taking alone is sufficient to make a diagnosis in 75% of patient encounters, before further reducing the number of clinical differentials through physical examination and additional tests [1]. However, the amount of time available to acquire an appropriate patient history is decreasing. The increasing demand and administrative burden on healthcare services have resulted in physician-patient contact time becoming shorter. A study in the United States highlighted that physicians spent 27% of their total time on direct clinical face time with patients and 49.2% of their time on the electronic health record (EHR) and deskwork [2]. During in-person consultation, on average, only 52.9% of physicians’ time was spent on direct clinical face time and 37.0% on EHR and deskwork [2]. The consultation length is directly associated with better health outcomes, fewer prescriptions, and better recognition of long-term and psychosocial problems [3,4]. On the other hand, a shorter consultation time has been associated with overuse of antibiotics, polypharmacy, and poor communication with patients [5,6]. Whether this is attributable to diagnostic uncertainty or fear of medicolegal repercussions remains to be evaluated.

### CAHT vs In-Person Interviews

Considering restrictions to consultation time and the associated negative consequences, patient-provided health data outside of consultation can prove invaluable in health care delivery. Several health bodies, including the Centers for Disease Control and Prevention, advocate for the serial collection of health surveys in routine clinical care [7]. These have traditionally been completed with pen and paper and are commonly not collected due to numerous barriers including difficulties in logistics of acquisition, distribution, and collection of paper forms; difficulties in understanding and completing surveys by patients; the potential disruption of clinic workflow; difficulties in interpreting results; lack of perceived clinical relevance; and cost (materials, manpower, and distribution) [8].

A CAHT system (CAHTS) is a digital tool that aids clinicians in gathering data from patients through health surveys to inform a diagnosis or treatment plan [9]. The benefits of CAHTS are evident in the potential time saving in terms of acquiring the patient history outside of consultation, reducing the administrative burden of entering this information, increasing patient face-to-face time, and leveraging these data through medical records using machine learning algorithms for decision support. Patients have reported high satisfaction from helping their physician through the completion of interactive computerized interviews [10]. CAHT is an effective strategy to empower patients to be active in their own care (ie, patient engagement) [11]. Increased patient involvement results in improved participation in personal care, compliance with medication, adherence to recommended treatment, and monitoring of prescriptions and doses (for a complete overview of potential advantages, see Textbox 1).

**Potential advantages of computer-assisted history taking (CAHT) [9,12-16].**

- Enables history taking prior to or post consultation
- Enables completion at the patient’s pace without feeling rushed
- High compliance rate (for patients)
- Time efficient (for both patient and clinician)
- Collects complete and accurate patient data
- Provides legible summaries
- Less likely to have falsified data
- Patients reveal more sensitive “private” and social information
- Patients are better prepared for the medical interview
- Reduces staff labor costs
- Reduces diagnostic error
- Enables remote completion
- Allows health care professionals to make additional entries or changes
- Allows for seamless integration into the patient electronic health record (EHR)
- Can incorporate artificial intelligence and decision support systems
- Can prompt educational messages or modules

CAHT can be done remotely through mobile devices at a time and place convenient to the patient. Furthermore, CAHT can improve the comprehensiveness of history taking by standardizing algorithms and extending the level of questioning through branching logic based on participant responses. Research has shown that clinicians are ineffective at acquiring comprehensive patient histories; they tend to miss 50% of psychosocial and psychiatric problems and do not elicit 54% of patient health problems [9].

Perhaps one of the greatest advantages of CAHTS is providing a shared knowledge base of clinical presentations and outcomes that is readily accessible to all providers. Decision making, especially in medicine, is complex and multifaceted. The accuracy of decisions is directly linked to whether the relevant information is readily available from memory [17]. One doctor’s decision process is directly linked to personal experience and whether that knowledge is readily retrievable (ie, comes to mind or is accessible at the time from sources such as textbooks, online libraries). In contrast, a digitized database allows for combined case histories, including multiple different variables (eg, lab results, imaging) to be stored, processed, and rapidly accessed to support decision making. This could greatly facilitate diagnostic accuracy, especially in rarer diagnostic cases.

### Overcoming Limitations to CAHT

**Figure 1:**
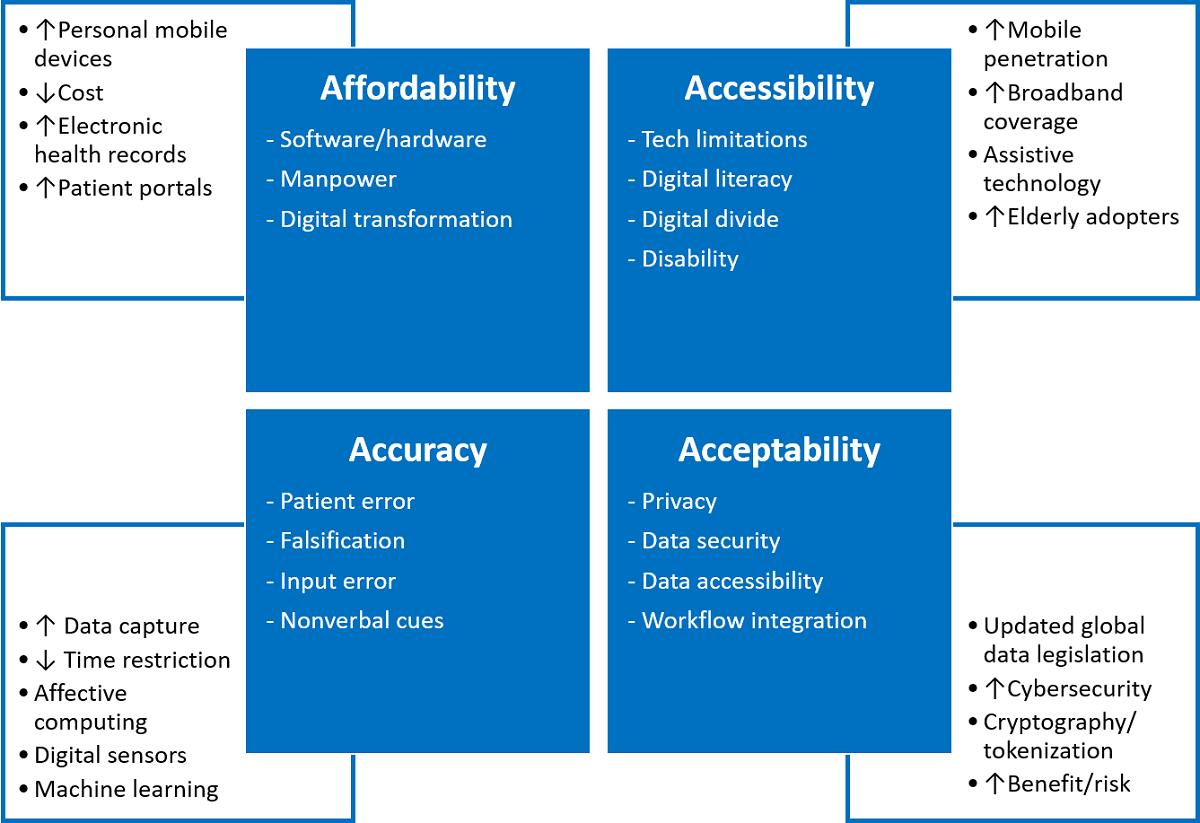
Overview of barriers to implementation of computer-assisted history taking systems (CAHTS) and changing landscape and technological solutions.

It is clear that there are many advantages to CAHT; however, its limitations could explain the slow rates of adoption of patient-provided health information in general [18]. We have grouped these limitations under 4 themes identified through literature reviews: accessibility, affordability, accuracy, and acceptability. We subsequently discuss how technological advances offer solutions to overcoming these (see Figure 1).

#### Accessibility

During the inception of CAHT in 1960, technology represented a potential barrier to implementation due to accessibility to computers. However, since then, innovation in mobile computing devices has seen a proliferation in personal devices from PDAs, followed by smartphones and tablets. Mobile devices have become ubiquitous accessories with more mobile devices on the planet now than people [19]. The digital divide between low- and middle-income countries and high-income countries is progressively narrowing, as mobile phone penetration rates and mobile broadband and internet access continue to expand [20]. The number of smartphone users globally has just surpassed 3 billion [21], with penetration rates that range from around 24% in India to 90% in high-income countries [20]. Hence, with computing power, communications, and internet now being untethered to physical, on-premise computers, access is no longer a limiting factor but rather facilitates the ability to deliver CAHT.

The early adopters of technology, including mobile phones, are commonly the young, tech-savvy, and able-bodied persons, previously limiting those who are older, digitally naïve, and disabled (hearing or visually disabled) from utilizing them. However, the elderly are increasingly becoming avid adopters of technology. In the United States, the fastest growing segment of smartphone adopters are aged 44-75 years old with a compound annual growth rate of close to 8% from 2015-2017 [22]. Mobile devices are being designed with assistive technology to further facilitate use. In mobile phones, for example, there are now a variety of accessibility options available, including text-to-speech output, screen magnification, audio amplifiers, hearing aid compatibility, and hands-free operation [23], facilitating use by those with visual or hearing disabilities. Assistive technology is also leveraging embedded systems and wearables. A “smart glove” is one such example, which recognizes basic hand gestures and converts them into speech or text [24]. Wearables allow for the passive collection of patient data, both physiological and pathological information, continuously and in real time, directly into EHRs. This reduces the barrier to acquiring data actively and can be used in health and safety monitoring, chronic disease management, disease diagnosis and treatment, and rehabilitation [25]. These data points could further augment CAHT; however, further research would need to be done to evaluate this benefit.

Beyond assistive technology, CAHT can be delivered in multiple formats including visually rich media, audible questionnaires, and multiple languages, tailoring the content to the targeted participant group. In terms of user accessibility, therefore, it is increasingly becoming ubiquitous and accessible by all.

The great strides taken to digitize health data and integrate EHRs have resulted in a highly fragmented system wherein health data are distributed in silos across the continuum of care with limited accessibility between providers and systems. Lack of interoperability and access to data therefore limit the potential benefits of CAHT. As in other industries, the barriers to interoperability are generally not technological but cultural and require the close coordination and collaboration of various stakeholders, including patients, providers, software vendors, legislators, and information technology professionals [26]. Over the last few years, there have been a number of improvements in policy, alignment of incentives, and the wider adoption of data-sharing protocols and infrastructure to help mitigate these accessibility issues. A number of health information technology solutions, such as health information exchanges, have propagated the collaboration of health systems to integrate across many different digital silos [27].

#### Affordability

Although CAHTS are viewed as cost-effective compared to regular standard of care, few studies have rigorously assessed the cost-benefit. Of those that have, most of the data available are related to changes in utilization of health care services due to health information technology [28]. There is a number of cost factors that can be taken into account, including equipment, time, manpower, and other costs. In practice, a CAHTS can be integrated into an EHR to allow for a data record and to leverage data insights accumulated from multiple sources, including lab results, imaging, and doctors’ records. In isolation, the benefits of a CAHT could not be fully realized. Hence, equipment costs when considering deploying a health information system or EHR can be significantly high. These costs can be categorized as system costs and induced costs [29]. System costs include the costs of software and hardware, training, implementation, ongoing maintenance, and support. Induced costs are related to the temporary productivity loss during EHR implementation. Several studies estimate the cost of purchasing and installing an EHR ranging from US $15,000 to US $70,000 per provider [30] with subsequent yearly maintenance costs. Previously, these costs may have presented a strong financial barrier to implementing CAHTS; however, EHR adoption rates have vastly increased. From 2008 till 2017, office-based physician adoption of EHRs in the United States has more than doubled, from 42% to 86% [31], with nearly universal adoption in the United Kingdom, Netherlands, Australia, and New Zealand [32]. Hence, considering the widespread prevalence of EHRs and initial sunk costs, the additional cost of implementing CAHTS is very low. Three of the largest EHR vendors (Epic, Allscripts, and Cerner) [33] all offer the capability to send questionnaires to patients and to receive structured data directly into the medical record. Although cost barriers to implementation have been greatly reduced over the past decades, a comprehensive analysis would need to be done to evaluate actual costs.

The cost of accessing CAHTS and health care systems outside of premises is also borne by the patients. The biggest factor limiting online access is cost, with the internet and mobile phones still not affordable for many around the world. Two and half billion people live in countries where the cost of the cheapest available smartphone is >25% of the average monthly income [34]. A number of organizations are seeking to improve these numbers, including the Web Foundation, the United Nations, and other national governments, which has been further necessitated by the COVID-19 pandemic. A part of the United Nation's Sustainable Development Goals is to achieve “universal access” in the least developed countries by the end of 2020. The Web Foundation defines “universal access” as 85% of a country's population [35].

#### Accuracy

Previously, there was some skepticism regarding the accuracy of CAHT in the sense that patients may not provide accurate information through a digital survey as opposed to in-person consultation with a physician. Postulated reasoning for this includes patient’s failure to read questions and answers carefully, misunderstood questions, mistaken selection of answers, failure to comprehend prior diagnoses, and intentional entry of false information [36]. The accuracy of patient’s answers in terms of mistaken entries, not deliberate falsification of inputs, is a significant issue for both computerized and physician history taking [36]. Provider errors beyond communication barriers can occur in patient notetaking, including incomplete patient notes and illegible handwriting, as well as errors in data input or transcription into EHRs. An analysis of medical malpractice cases found that incorrect information (eg, faulty data entry) was the top EHR-related contributing factor, contributing to 20% of reviewed cases [37]. Essentially, human entry error will always be a factor, both on the patient and provider side; however, false information can potentially be reduced through CAHTS. There is substantial evidence to support that direct reporting of symptoms by patients through CAHTS more accurately reflects their health status than through clinician elicitation [38]. Face-to-face interview methods typically result in reporting of lower rates of socially sensitive risk behaviors compared to self-administered questionnaires, attributable to social desirability bias [39]. For example, patients are more likely to report sensitive information including intimate partner violence, elective abortions, and high-risk behavior such as smoking status in computer interviews [40]. The nature of the doctor-patient relationship and demographic (eg, different cultural norms, age, and gender) may also influence patient reporting [41]. Another source of error includes unintentional reporting error due to poor recall and situational or time pressure to respond (ie, the patient feels they are on the spot or the doctor is rushing them). The benefit to CAHT is that it is not confined to consultation time, therefore is not time restricted, and the questions may be algorithm-based allowing for a structured, comprehensive collection of data rather than ad hoc questioning dependent on the provider’s thought process.

CAHTS are inherently limited by the inability to record nonverbal communication. Computers are “unable to detect nonverbal behavior, for example, sense a patient’s mood which might easily be picked up in a consultation” [4]. However, technology has since evolved to be able to capture various nonverbal behavioral cues like facial expressions, vocalizations, postures, gestures, and appearance. There have been significant advances in the field of affective computing, which is the study and development of systems and devices that can recognize, interpret, process, and simulate human affects [42]. It is an interdisciplinary field that leverages the crosspollination of artificial intelligence (including speech processing, computer vision, and machine learning) and human sciences (psychology, anthropology, and sociology). By utilizing digital sensor data such as video or sound recordings, behavioral patterns can be evaluated. For example, microexpressions (involuntary, brief facial movements) play an essential part in understanding nonverbal communication and deceit detection [43]. Due to the nature of microexpressions being extremely brief and subtle, it is difficult to perceive with the naked eye. Advances in computer algorithms and video acquisition technology are rendering machine analysis of facial microexpressions an increasing possibility [44]. Facial expressions can be used in combination with other modalities including head and hand movements to detect deception [45].

Although there is still a long road to go towards accurate real-time assessment, we can now leverage several modalities to detect nonverbal cues while completing CAHT. Natural language processing (NLP) may offer a more readily available solution to deceit detection through text analysis. In recent years, with the explosive popularity of social media and subsequent exposure of fake news, increasing attention has been put on lie detection using artificial intelligence and deep learning techniques. Beyond falsification of information, NLP can analyze sentiment, which includes the words and symbols used in text to indicate positive and negative opinions, and emotions. Sentiment analysis has been well studied in health care, including its relation to outcomes (eg, greater positive sentiment within discharge summaries) associated with significantly decreased risk of readmission [46]. Through technology, we may therefore be able to predict mental state or falsification of information with potentially greater accuracy than by clinicians.

#### Acceptability

Despite the benefits of CAHTS, a key barrier to adoption could be acceptability to both patients and providers. Previous challenges included acceptability in terms of technology and associated challenges with its use [47]. This may have been the case in the past with unreliable platforms and outdated devices (eg, PDAs). However, studies have shown that patients report a high level of satisfaction with CAHT, with the majority (69%) believing that their medical care is enhanced by CAHTS [36]. Youth find computerized questionnaires “equally or more acceptable than the usual clinical interview or a written questionnaire” [9].

Other identified barriers to acceptability include cognition, motivation, and perception [48]. For example, one 2003 study of elderly persons with cognitive disabilities found that people did not use 15% of the devices they owned mostly because they did not fit their needs [49]. In order for CAHTs to be adopted, all stakeholders need to be involved in the design and development process. Research suggests that involving users in the design and development of a new system will improve the system’s quality and result in a higher level of user acceptance [50]. To this point, there have been an increasing number of initiatives focused on this approach to engage elderly. The Dutch National Care for the Elderly Programme was one such initiative that put the needs of elderly people at the heart of the program, ensuring their active participation to provide person-centered and integrated care better suited to their needs [51]. The European Commission has also set up a group of measures to improve e-accessibility for older people as part of the e-Inclusion policies [52]. Through user-centered design and educational training programs, accessibility and adoption can be greatly increased.

Considerations and barriers to use from a provider’s perspective include (1) availability of practice workflows and protocols related to patient-generated health data and (2) data storage, accessibility, and ease of use at the point of care [53].

#### Privacy and Data Security

Acceptability is also largely attributable to fears regarding privacy and data security. The concerns are often related to who has access to sensitive personal health information and to breaches from external malicious counterparts. To address data privacy concerns, there have been updates to global data protection legislation. The General Data Protection Regulation, enforced from 2018, is one such example that strengthens data protection for all individuals within the European Union. The Health Insurance Portability and Accountability Act, enacted in 1996, safeguards the collection, storage, and disclosure of identifiable health data in the United States [54]. Despite these regulatory changes, health care is one of the most data-rich industries globally, with approximately 30% of the world’s electronic data storage occupied by health care information [55]. A person is estimated to accrue more than 1 million gigabytes of health-related data in their lifetime through EHRs, digitized diagnostics, and wearable medical devices [55]. This deluge of health data and the potential value attributable to ransom or sales on the black market makes health data one of the most targeted sources to cyber threats. In 2018, health care data breaches accounted for 24% of all investigated breaches across all industries [56]. In response to this increasing cybercrime, the global cybersecurity market, estimated to be US $100 billion in 2017, is expected to grow to US $173 billion in 2022 at a compound annual growth rate of 11.6% [57]. New technologies are emerging to market to address these security concerns including cryptography (translating data into code, only accessible with an access key), tokenization (sensitive data are substituted with a randomly generated value or token), and distributed ledgers or blockchain (a distributed list of records or blocks that are linked using cryptography). Estonia is an example of an early adopter of this technology utilizing blockchain technology to secure health care data and process transactions [58]. This puts patients in control of their data and allows them to grant or restrict access to different groups including their health care provider, family members, or research teams. With greater control of personal data, there is a greater sense of security.

Although advances in technology are fortifying against data breaches, no system is infallible. The question should be whether the potential risk outweighs the perceived benefit. Logically, people are not willing to share personal information digitally, risking confidentiality, if there is no perceived benefit to them. Historically, this may have been true, as digital health data were largely collected, but underutilized. However, with advances in computing power and artificial intelligence, these volumes of data can be analyzed using predictive models, to provide more accurate and personalized health care. Artificial intelligence has been shown to effectively diagnose and predict multiple conditions by leveraging machine learning, NLP, and image recognition [59]. It has been shown to predict hospitalization due to heart disease roughly a year in advance with an accuracy of 82% [60]. The pervasive use and continuously improving algorithms employed by artificial intelligence in health care will result in greater realized benefit, largely outweighing the potential risks. The benefits of personalization over data privacy are evident in the consumer industry, where 57% of consumers are willing to share personal data in exchange for personalized offers or discounts [61]. Therefore, how much greater would the benefit of longevity and good health be?

### Conclusion

Patient health information is increasingly collected through multiple modalities, including EHRs, wearables, and connected devices. CAHT could provide an additional channel to collect highly relevant, comprehensive, and accurate patient information while reducing the burden on clinicians and face-to-face consultation time. Barriers to implementation and use in practice, such as accessibility, affordability, accuracy, and acceptability, may be addressed by advances in technology, computing power, and ubiquity of personal devices. Thus, perhaps there is no better time than now to adopt CAHT in standard care.

## Abbreviations

CAHT: computer-assisted history taking
CAHTS: computer-assisted history taking system
EHR: electronic health record
NLP: natural language processing

## References

[1] Ohm F, Vogel D, Sehner S, Wijnen-Meijer M, Harendza S (2013). Details acquired from medical history and patients' experience of empathy--two sides of the same coin. BMC Med Educ.

[2] Sinsky C, Colligan L, Li L, Prgomet M, Reynolds S, Goeders L, Westbrook J, Tutty M, Blike G (2016). Allocation of Physician Time in Ambulatory Practice: A Time and Motion Study in 4 Specialties. Ann Intern Med.

[3] Elmore N, Burt J, Abel G, Maratos FA, Montague J, Campbell J, Roland M (2016). Investigating the relationship between consultation length and patient experience: a cross-sectional study in primary care. Br J Gen Pract.

[4] Howie J, Porter AM, Heaney DJ, Hopton JL (1991). Long to short consultation ratio: a proxy measure of quality of care for general practice. Br J Gen Pract.

[5] Dugdale D, Epstein R, Pantilat SZ (1999). Time and the patient-physician relationship. J Gen Intern Med.

[6] Irving G, Neves AL, Dambha-Miller H, Oishi A, Tagashira H, Verho A, Holden J (2017). International variations in primary care physician consultation time: a systematic review of 67 countries. BMJ Open.

[7] (2016). Hearts: technical package for cardiovascular disease management in primary health care. World Health Organization.

[8] Williams CA (2004). Usability of a Computer-assisted Interview System for the Unaided Self-entry of Patient Data in an Urban Rheumatology Clinic. Journal of the American Medical Informatics Association.

[9] Pappas Y, Anandan C, Liu J, Car J, Sheikh A, Majeed A (2011). Computer-assisted history-taking systems (CAHTS) in health care: benefits, risks and potential for further development. Inform Prim Care.

[10] Arora S, Goldberg A, Menchine M (2014). Patient impression and satisfaction of a self-administered, automated medical history-taking device in the Emergency Department. West J Emerg Med.

[11] Ammenwerth E, Schnell-Inderst P, Hoerbst A (2011). Patient empowerment by electronic health records: first results of a systematic review on the benefit of patient portals. Stud Health Technol Inform.

[12] Wolford G, Rosenberg SD, Rosenberg HJ, Swartz MS, Butterfield MI, Swanson JW, Jankowski MK (2008). A clinical trial comparing interviewer and computer-assisted assessment among clients with severe mental illness. Psychiatr Serv.

[13] Tompkins BM, Tompkins WJ, Loder E, Noonan AF (1980). A Computer-Assisted Preanesthesia Interview. Anesthesia & Analgesia.

[14] Bachman J (2007). Improving care with an automated patient history. Fam Pract Manag.

[15] Bachman JW (2003). The patient-computer interview: a neglected tool that can aid the clinician. Mayo Clin Proc.

[16] Pappas Y, Wei I, Car J, Majeed A, Sheikh A (2011). Computer-assisted versus oral-and-written family history taking for identifying people with elevated risk of type 2 diabetes mellitus. Cochrane Database Syst Rev.

[17] Zimny NJ, Tandy CJ (1993). Development of a computer-assisted method for the collection, organization, and use of patient health history information in physical therapy. J Orthop Sports Phys Ther.

[18] Ancker J, Mauer E, Kalish R, Vest J, Gossey J (2019). Early Adopters of Patient-Generated Health Data Upload in an Electronic Patient Portal. Appl Clin Inform.

[19] Radicati S (2014). Mobile Statistics Report, 2014-2018. The Radicati Group, Inc.

[20] Wiggers K (2019). Pew: Smartphone penetration ranges from 24% in India to 95% in South Korea. VentureBeat.

[21] Smartphone users worldwide 2016-2021. Statista.

[22] Global mobile consumer trends: Second edition. Deloitte.

[23] Manduchi R, Coughlan J, Miesenberger K, Klaus J, Zagler W, Karshmer A (2008). Portable and Mobile Systems in Assistive Technology. Computers Helping People with Special Needs. ICCHP 2008. Lecture Notes in Computer Science, vol 5105.

[24] Sarji DK (2008). HandTalk: Assistive Technology for the Deaf. Computer.

[25] Lu L, Zhang J, Xie Y, Gao F, Xu S, Wu X, Ye Z (2020). Wearable Health Devices in Health Care: Narrative Systematic Review. JMIR Mhealth Uhealth.

[26] Reisman M (2017). EHRs: The Challenge of Making Electronic Data Usable and Interoperable. P T.

[27] Gamache R, Kharrazi H, Weiner J (2018). Public and Population Health Informatics: The Bridging of Big Data to Benefit Communities. Yearb Med Inform.

[28] Chaudhry B, Wang J, Wu S, Maglione M, Mojica W, Roth E, Morton SC, Shekelle PG (2006). Systematic review: impact of health information technology on quality, efficiency, and costs of medical care. Ann Intern Med.

[29] Wang SJ, Middleton B, Prosser LA, Bardon CG, Spurr CD, Carchidi PJ, Kittler AF, Goldszer RC, Fairchild DG, Sussman AJ, Kuperman GJ, Bates DW (2003). A cost-benefit analysis of electronic medical records in primary care. The American Journal of Medicine.

[30] (2014). How much is this going to cost me?. The Office of the National Coordinator for Health Information Technology (ONC).

[31] (2020). Electronic Medical Records/Electronic Health Records (EMRs/EHRs). Centers for Disease Control and Prevention.

[32] Schoen C, Osborn R, Squires D, Doty M, Rasmussen P, Pierson R, Applebaum S (2012). A survey of primary care doctors in ten countries shows progress in use of health information technology, less in other areas. Health Aff (Millwood).

[33] (2013). The top 100 EHR companies (Part 1 of 4). Medical Economics.

[34] (2020). Mobile devices are too expensive for billions of people — and it’s keeping them offline. Alliance for Affordable Internet.

[35] (2015). The 17 Goals. United Nations Department of Economic and Social Affairs.

[36] Zakim D, Braun N, Fritz P, Alscher MD (2008). Underutilization of information and knowledge in everyday medical practice: evaluation of a computer-based solution. BMC Med Inform Decis Mak.

[37] Zhou L, Blackley SV, Kowalski L, Doan R, Acker WW, Landman AB, Kontrient E, Mack D, Meteer M, Bates DW, Goss FR (2018). Analysis of Errors in Dictated Clinical Documents Assisted by Speech Recognition Software and Professional Transcriptionists. JAMA Netw Open.

[38] Chung A, Basch EM (2015). Incorporating the patient's voice into electronic health records through patient-reported outcomes as the "review of systems". J Am Med Inform Assoc.

[39] Islam MM, Topp L, Conigrave KM, van Beek I, Maher L, White A, Rodgers C, Day CA (2012). The reliability of sensitive information provided by injecting drug users in a clinical setting: clinician-administered versus audio computer-assisted self-interviewing (ACASI). AIDS Care.

[40] Mears M, Coonrod DV, Bay RC, Mills TE, Watkins MC (2005). Routine history as compared to audio computer-assisted self-interview for prenatal care history taking. J Reprod Med.

[41] Schouten BC, Meeuwesen L (2006). Cultural differences in medical communication: a review of the literature. Patient Educ Couns.

[42] Tao J, Tan T, Tao J, Tan T, Picard RW (2005). Affective Computing: A Review. Affective Computing and Intelligent Interaction. ACII 2005. Lecture Notes in Computer Science, vol 3784.

[43] Borza D, Danescu R, Itu R, Darabant A (2017). High-Speed Video System for Micro-Expression Detection and Recognition. Sensors (Basel).

[44] Oh Y, See J, Le Ngo AC, Phan RC, Baskaran VM (2018). A Survey of Automatic Facial Micro-Expression Analysis: Databases, Methods, and Challenges. Front Psychol.

[45] Meservy T, Jensen M, Kruse J, Twitchell D, Tsechpenakis G, Burgoon J, Metaxas D, Nunamaker J (2005). Deception Detection through Automatic, Unobtrusive Analysis of Nonverbal Behavior. IEEE Intell. Syst.

[46] Gohil S, Vuik S, Darzi A (2018). Sentiment Analysis of Health Care Tweets: Review of the Methods Used. JMIR Public Health Surveill.

[47] Wei I (2011). Computer-assisted versus oral-and-written dietary history taking for diabetes mellitus. Cochrane Database Syst Rev(12): p. Cd00.

[48] Wildenbos G, Peute L, Jaspers M (2018). Aging barriers influencing mobile health usability for older adults: A literature based framework (MOLD-US). Int J Med Inform.

[49] Mann WC, Goodall S, Justiss MD, Tomita M (2002). Dissatisfaction and nonuse of assistive devices among frail elders. Assist Technol.

[50] De Vito Dabbs A, Myers BA, Mc Curry KR, Dunbar-Jacob J, Hawkins RP, Begey A, Dew MA (2009). User-centered design and interactive health technologies for patients. Comput Inform Nurs.

[51] Jong BM, Wynia K, Geluk-Bleumink A (2018). Ageing Better in the Netherlands.

[52] (2007). 52007SC1469 - EN. EUR-Lex.

[53] Cohen DJ, Keller SR, Hayes GR, Dorr DA, Ash JS, Sittig DF (2016). Integrating Patient-Generated Health Data Into Clinical Care Settings or Clinical Decision-Making: Lessons Learned From Project HealthDesign. JMIR Hum Factors.

[54] Gostin LO, Halabi SF, Wilson K (2018). Health Data and Privacy in the Digital Era. JAMA.

[55] Sensmeier J (2017). Harnessing the power of artificial intelligence. Nurs Manage.

[56] Jalali MS, Razak S, Gordon W, Perakslis E, Madnick S (2019). Health Care and Cybersecurity: Bibliometric Analysis of the Literature. J Med Internet Res.

[57] Annex A-3: Cyber Security. Infocomm Media Development Authority.

[58] Healthcare. e-estonia.

[59] Topol EJ (2019). High-performance medicine: the convergence of human and artificial intelligence. Nat Med.

[60] Dai W, Brisimi TS, Adams WG, Mela T, Saligrama V, Paschalidis IC (2015). Prediction of hospitalization due to heart diseases by supervised learning methods. Int J Med Inform.

[61] (2018). Digital Advertising 2020. Salesforce.

